# Fish: a new xenograft source for maxillary sinus lifting^*^

**DOI:** 10.1590/1678-7757-2024-0245

**Published:** 2024-11-22

**Authors:** Emrah Soylu, Musab Süleyman Kilavuz, Fatih Duman, Hasan Ekeer, Zeynep Burçin Gönen, Beyza Kahraman, Arzu Hanım Yay, Demet Bolat

**Affiliations:** 1 Erciyes University Faculty of Dentistry Department of Oral and Maxillofacial Surgery and DentBioChem Biotechnology Co. Kayseri Türkiye Erciyes University Faculty of Dentistry, Department of Oral and Maxillofacial Surgery and DentBioChem Biotechnology Co., Erciyes Teknopark, Kayseri, Türkiye.; 2 Erciyes University Faculty of Science Department of Hydrobiology Kayseri Türkiye Erciyes University, Faculty of Science, Department of Hydrobiology, Kayseri, Türkiye; 3 Erciyes University Faculty of Dentistry Research Laboratory Kayseri Türkiye Biologist, Erciyes University, Faculty of Dentistry, Research Laboratory, Kayseri, Türkiye; 4 Erciyes University Faculty of Medicine Department of Histology and Embryology Kayseri Türkiye Erciyes University, Faculty of Medicine, Department of Histology and Embryology, Kayseri, Türkiye

**Keywords:** Hydroxyapatite, Fish-derived hydroxyapatite, Autologous graft, Maxillofacial surgery, Maxillary sinus floor augmentation, Rabbits

## Abstract

**Objective:**

Although autogenous grafting is accepted as the gold standard in intraoral grafting, xenogenous grafts are frequently used in sinus lift surgeries due to their osteoinductive and osteoconductive properties. This study aimed to investigate the efficacy of fish spine-derived xenogenic grafts in sinus augmentation surgery.

**Material and Methods:**

In this study, a fish spine-derived xenogenic graft was produced for comparison with autogenous graft and bovine derived xenogenic grafts. Twenty-one New Zealand rabbits were used. Autogenous grafts (AG- Group 1), as well as bovine-derived (bHAP - Group 2) and fish spine-derived (fHAP - Group 3) xenogenic grafts were placed in the right and left sinuses of rabbits. The animals were sacrificed at the 4th and 8th weeks. New bone formation (NBF) was evaluated through histological examination, while bone volume (BV), new bone surface/bone volume (BS-BV), new bone surface/tissue volume (BS-TV), and trabecular separation (Tb-Sp) were assessed via Micro-CT. Statistical significance was considered at p<0.05.

**Results:**

Histological examination revealed a significant difference in NBF between AG-bHAP (p<0.001), as well as between fHAP-bHAP (p<0.001) in the fourth-week group. No significant difference was found in the eighth-week group (p=0.130). In the eighth-week group, a statistically significant difference was found between fHAP and bHAP in terms of BV. (p=0.007).

**Conclusion:**

Although both graft materials used in this study showed positive effects on bone regeneration, fHAP and AG presented similar effects on bone regeneration and were superior to bHAP.

## Introduction

The bone volume of the maxillary posterior area can be limited or inadequate for implant placement due to traumatic tooth extraction, physiological bone resorption or excessive sinus pneumatization.^1^ Additionally, poor bone quality resulting from low bone density and thinning of the cortical bone can negatively affect the primary stability and survival of dental implants.^2–3^ Bone augmentation procedures are crucial to overcoming these limitations. The most common procedure for vertical bone augmentation in the maxillary posterior area is called "maxillary sinus grafting" or the "lateral window technique". This technique was first described by Tatum and presented by Boyne and James in 1980.^4^

Autologous bone grafting (AG) is considered the gold standard due to its ideal biological properties, including osteogenic, osteoinductive, and osteoconductive properties.^1–3^ Despite these advantages, AG also has several drawbacks, such as the need for a new surgical site, donor site morbidity, and a limited amount of obtainable graft material.^3^

Various grafting materials, such as freeze-dried bone, demineralized freeze-dried bone, and xenogenic or synthetic bone substitutes, have been used as alternatives to AG.^5^ Stem cell therapies and tissue engineering have also gained popularity in the regeneration of bone defects.^6^ Furthermore, combining bone substitutes with recombinant human bone morphogenetic protein type 2 (rhBMP-2) has attracted increasing interest.^7^

Xenografts have gained popularity as a solution to the limitations of the AG.^8–10^ Generally, xenografts are derived from bovine sources in an inorganic form, though porcine and equine-derived materials are also available.^10–12^ Xenografts have excellent bioactivity, mechanical stability, and angiogenic properties, while also lacking toxicity and inflammatory and antigenic reactions.^13,14^ However, it was histologically shown that their potential for new bone formation is limited.^2^

Fish spine, a natural byproduct, can also serve as source for fish-derived xenografts. Like other xenografts, they are based on calcium phosphate and consist of hydroxyapatite (HAP), which is responsible for their osteoconductive properties.^13–16^ *In vivo* and *in vitro* studies on fHAP showed promising results in terms of bone regeneration.^2^ Granito, et al.^13^ (2018) reviewed the literature on fHAP and concluded that it is non-cytotoxic and suitable for biomedical applications. Venkatesan, et al.^17^ (2015) reported that fHAP positively interacted with mesenchymal stem cells (MSCs) and Prathibha, et al.^18^ (2024) showed that the fish scale-derived HAP have potential for bone regeneration in socket preservation, allowing stem cell migration and proliferation.

Several studies have investigated the effect of fHAP from different fish species on bone healing *in vitro*. To the best of our knowledge, no *in vivo* studies have investigated fish spine-derived HAP. Hence, this study aimed to evaluate the effect of fish spine-derived HAP on bone healing in an experimental sinus-lifting model in rabbits.

## Methodology

### Experimental procedure

The study protocol was approved by the Local Ethical Committee of the Animal Research at Erciyes University (04.12.2019/225) and conducted under the terms of the Animal Research: Reporting *In Vivo* Experiments (ARRIVE) 2.0 protocol. The study involved 21 healthy, previously unused, genetically unmodified New Zealand female rabbits, aged 6 to 12 months, weighing between 2.5 and 3.5 kg. The animals were housed in a Good Clinical Research facility at Erciyes University, individually placed in stainless-steel cages, and kept at a temperature of 22 ±1°C, with 50% humidity, and a 12-hour light-dark cycle.

### Power analysis

According to the power analysis performed using G*Power software, based on bone fraction volume values obtained at the fourth week after the application in the study of Sununliganon, et al.^2^ (2014), which examined the osteogenic effect of grafting for maxillary sinus lifting, with alpha=0.05, 1-B=0.90 (90% power) and d=1.973 effect size, it was determined that at least six samples were required in each group. Considering possible losses and infections, the sample size was increased by 10%, resulting in seven samples per group.

### Surgical procedure

All animals were placed under general anesthesia with a combination of ketamine 0.3 ml/kg (Alfamine, 50 mg/mL, Atafen, İzmir, Türkiye) and xylazine 0.25 mg/kg (Alfazyne, 20 mg/mL, Atafen, İzmir, Türkiye). The dorsal area of each rabbit was shaved and prepared with povidone-iodine solution and 0.5 mL of articaine (Ultracaine D-S, Sanofi Aventis, Istanbul, Türkiye) with 1:100,000 epinephrine injected subcutaneously along the midline of the nasal dorsum. The surgical fields were covered with a sterile cover.

**Figure 1:**
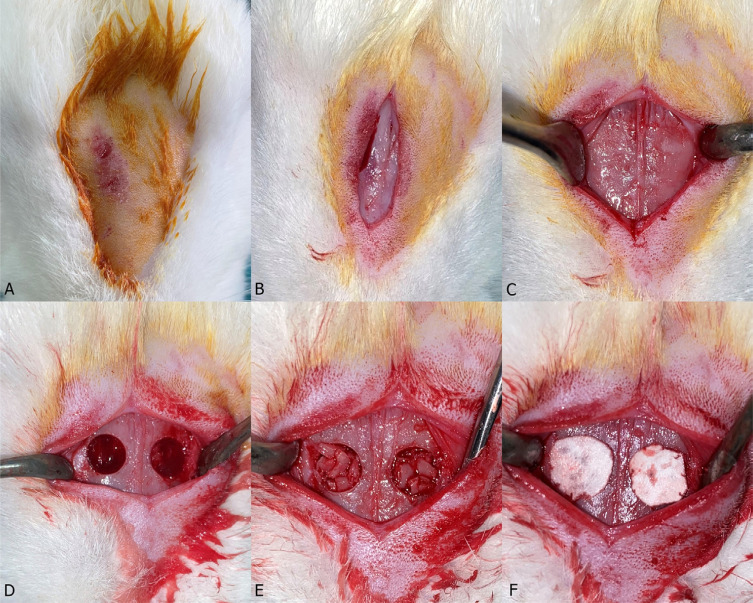
Intraoperative view. a) Nasal dorsum of the rabbit prepared for surgery. b) A mucoperiosteal incision was made. c) The mucoperiosteal flap was elevated and retracted to expose the nasal dorsum. d) Osteotomies were performed using a 5 mm diameter trephine burr, and the bony windows were removed. Sinus membranes were gently elevated with membrane elevators. e) fHAP and bHAP were placed. f) Collagen membranes were applied.

As described by Yılmaz, et al.^9^ (2017), a midline incision was made through the skin of the nasal dorsum to expose nasal bones and a naso-incisal suture line. A 5 mm trephine burr was used under continuous sterile saline irrigation to create symmetrical windows on both sides of the naso-incisal suture. The bony wall was gently elevated to avoid perforating the sinus membrane, and the sinus membrane was subsequently elevated using blunt-ended sinus lift curettes to place the graft materials. In Group 1 (AG), the bony window created before the sinus lifting procedure was crushed with a rongeur forceps, and the particulated AG was placed in the sinus cavity. In Group 2 (fHAP), a fish-derived xenogenic graft (particle size 0.25 to 1 mm) was used, and in Group 3 (bHAP), a bovine-derived xenogenic graft (particle size 0.25 to 1 mm) (MedPark, Busan, South Korea) was placed into the sinus cavity. All graft materials were weighed with precision scale, and 0.1 g of graft material was placed in each group. All surgical sites were covered with a resorbable collagen membrane (MedPark, Busan, South Korea). The periosteum and skin were closed using 3/0 Vicryl sutures (Figure 1). Postoperatively, a combination of intramuscular analgesics (1 mg/kg diclofenac sodium, Dikloron 75 mg/3 mL IM, Deva, Istanbul, Türkiye) and antibiotics (50 mg/kg cefazolin sodium, Cefamezin IM, Eczacıbaşı, Istanbul, Türkiye) was administered 6 and 8 hours separately. The surgical site was covered with topical antiseptics for three days. As per Yılmaz et al. and to avoid increasing the number of animals, no control group was used.

### Group assignment

A set of 42 notepapers was equally divided, with "left" written on half and "right" on the other, then placed in a bag. Another set of 42 notepapers with "AG", "fHAP" and "bHAP" (14 for each group) were placed in a separate bag. Before each rabbit was operated, the author BB drew a paper of from each bag to determine which type of graft would be placed in the left or right sinus. The animals’ ears were marked with tattoo ink as a group indicator, and a name/group tag was placed on the cage. Each group was divided into two subgroups. Three rabbits in each group were sacrificed at the end of the fourth week (28 days), and four were sacrificed at the end of the eighth week (56 days).

### fHAP preparation

#### Preparation for decomposition

The spine bones of Steelhead fishes (*Oncorhynchus mykiss*) were dissected and boiled in 100°C water for 1 hour. After removing the tissue remnants, the bones were placed in NaOH (1%) and acetone (solid-liquid rate 1:50) for 24 hours and washed with ultrapure water to remove protein, oil, and other organic compounds. Bone particles were placed in an oven and dried at 60°C for 6 hours. After drying, the bone fragments were crushed and turned into small particles, which were sieved and classified according to size.

#### Thermal decomposition

Calcination was performed in a high-heat oven. The bone particles were placed into the oven, with temperature increments set at 10°C/min, and exposed to 650°C heat for five hours.

#### Characterization of bone graft

The morphological features of the bone graft were examined with a Field Emission Scanning Electron Microscope (FE-SEM), and the chemical composition was analyzed with Energy Dispersive Spectroscopy (EDX). Trace element analysis was performed with Inductively Coupled Plasma Mass Spectrometry (ICP-MS), the crystal structure of the bone graft was detected with X-ray Powder Diffraction (XRD), surface area and porosity analysis was performed using BET theory, and functional group analysis was carried out using Fourier Transform Infrared Spectroscopy (FT-IR).

### Histological examination

Tissue samples were fixed in 10% buffered formalin for three days and decalcified in 10% formaldehyde and nitric acid solution for four weeks. After decalcification, the samples were dehydrated through ascending concentrations of ethyl alcohol, cleared with xylene, and embedded in paraffin. The samples were coronally sliced into 5 μm thick sections using a microtome (Leica RM 2155; Leica Instruments, Nussloch, Germany). From the 2000 to 3000 sections obtained from each tissue block, 40 to 45 sections per animal were selected for volumetric estimation using systematic random sampling, starting from a randomly selected sample and selecting every 50^th^ section. Selected sections were stained with hematoxylin-eosin (H&E) and Masson Trichrome (MT), evaluated by different blinded researchers under a light microscope at 100× and 400× magnifications (Olympus BX51, Tokyo, Japan). Photographs were taken using a color digital camera attachment (Microbrightfield, Williston, VT). Sections were also examined histopathologically to evaluate general structures and bone formation. The ImageJ software (NIH, USA) was used to evaluate five non-overlapping fields of each group's stained sections. New bone formation (NBF) was calculated in square micrometers using the same software.

### Micro-Ct examination

Tissue samples were dried after fixation and scanned using a micro-CT system (Bruker SkyScan 1275, Belgium) with a pixel size of 20 μm, tube voltage of 100 kVp, beam current of 100 μA, and a 0.5 mm Al/Cu filter. The detector's air calibration was performed before each scanning to minimize the presence of artifacts. Each sample was rotated 360° within an integration time of 4 min. The mean scanning time was approximately 2 h.

**Figure 2:**
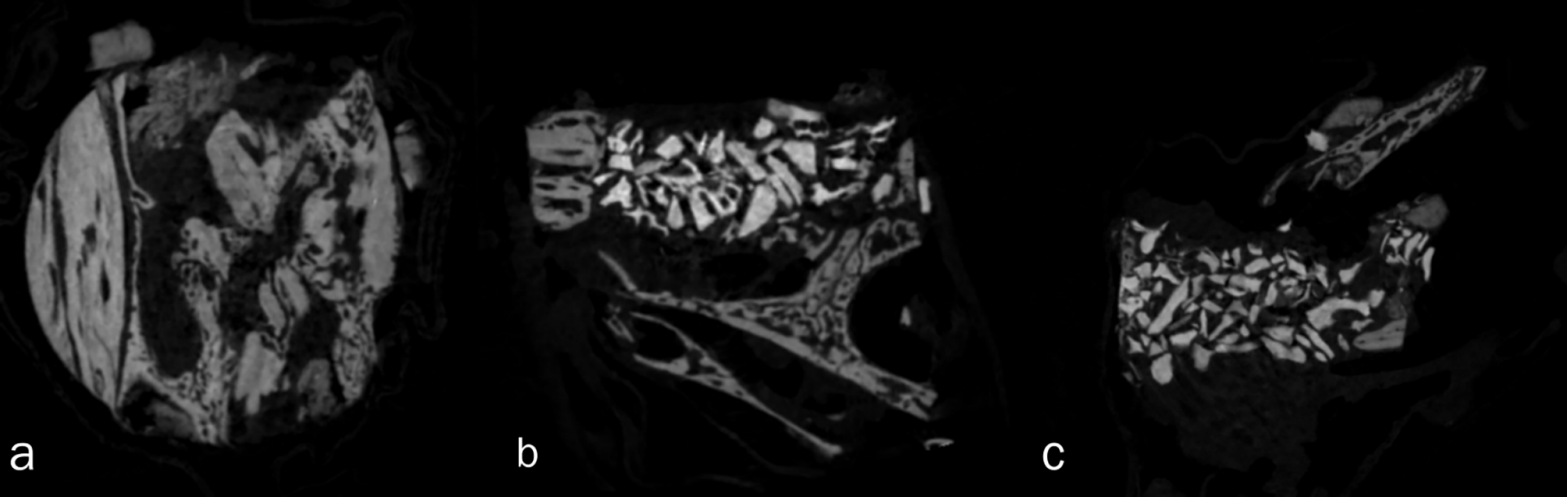
Micro CT images of the graft sites. A) Autologous graft, B) fHAP, C) bHAP

**Figure 3:**
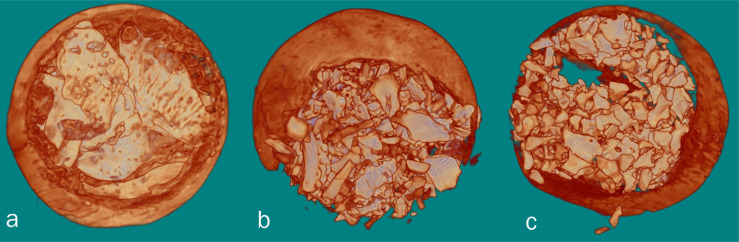
3D reconstruction of the Micro CT images. a) Autologous graft, b) fHAP, c) bHAP

The NRecon software (Version 1.6.7.2, Skyscan) was used for visualization and quantitative measurements of the samples, using the modified algorithm described by Feldkamp, et al.^19^ (1989) to obtain axial two-dimensional, 1000×1000-pixel images. (Figure 2) For the reconstruction parameters, ring artifact correction and smoothing were fixed at 0, and beam artifact correction was set at 40%. Contrast limits were applied following Skyscan instructions. 3D images were reconstructed with the CTVol v.2.2.1 software (Bruker, Kontich, Belgium), (Figure 3) and data analyses were performed using the CTAn v.1.12 software (Bruker, Kontich, Belgium). Bone mineral density (BMD), new bone volume (BV), total bone volume (TBV), tissue volume (TV), BV/TV, new bone surface (BS), BS/BV, BS/TV, trabecular thickness (TbTh), trabecular number (TbN), trabecular separation (TbSp) values were calculated.

### Statistical analysis

A web-based analysis program (Turcosa, www.turcosa.com.tr, Turcosa Analytic, Kayseri, Türkiye) was used for statistical analysis. The Shapiro-Wilk test was applied to evaluate normal distribution. Parametric results were analyzed using ANOVA, and non-parametric results were obtained using the Kruskal-Wallis test. Tukey HSD was used to determine statistical differences among groups, with p<0.05 considered statistically significant.

## Results

One rabbit was excluded due to death, and a total of 20 rabbits were included. One sinus was excluded due to infection, leaving a total of 39 sinuses (19 sinuses in the fourth-week group and 20 sinuses in the eighth-week group; 13 sinuses for AG, 13 sinuses for bHAP and 13 sinuses for fHAP) included in the study.

### fHAP characterization

#### FE-SEM and EDX analysis

The result of the FE-SEM analysis for surface morphology of fish-derived xenogen hydroxyapatite (fHAP) revealed a rough texture with nano- and microstructures, characterized by an irregular amorphous shape. Pore formations of various sizes were also observed, contributing to an increased surface area, which enhances interaction in biological environments or with other materials. The EDX spectrum analysis further verified that fHAP primarily consists of calcium (Ca), phosphorus (P), and oxygen (O), the three key elements required for hydroxyapatite, Ca_10_(PO_4_)_6_(OH)_2_ formation. It is a biomaterial widely used for bone tissue engineering due to its resemblance to the mineral components of natural bones. The comparison between fHAP and bHAP indicated similar elemental compositions, suggesting that the functionalization process did not significantly alter the base chemical composition of hydroxyapatite. However, surface roughness and porosities were observed in the FE-SEM micrographs, potentially influencing structural properties.

#### FT-IR analysis

FT-IR was conducted to further characterize the fHAP and verify the presence of functional groups specific to hydroxyapatite. The characteristic peaks for fHAP include: 1018.4 cm^−1^: Asymmetric stretching vibrations of the phosphate (PO_4_^3−^), a key identifier of hydroxyapatite. 601.3 cm^−1^ and 543.8 cm^−1^: Bending vibrations of the O-P-O bonds of the phosphate group, typical of hydroxyapatite; it also verifies the phosphate groups in the fHAP sample. Similar peaks were found in bHAP: 560.88 cm^−1^, 599.25 cm^−1^, 543.8 cm^−1^ and 601.3 cm^−1^: bending vibrations of the phosphate groups. Also of note are the 963.80 cm^−1^ and 1026.6 cm^−1^ peaks, which represent stretching vibrations of phosphate groups, thereby confirming the presence of hydroxyapatite. Additional peaks, such as 871.36 cm^−1^ and 1411.2 cm^−1^, suggest carbonate substitutions into the hydroxyapatite lattice, typical of biological apatites, giving bHAP a biomimetic character. The FT-IR peaks of fHAP and bHAP coincide well with those of hydroxyapatite, proving the existence of the compound in these two samples. The resemblance between the FT-IR spectra of fHAP and bHAP confirms that functionalization did not alter the general structure of hydroxyapatite, particularly the phosphate groups responsible for bioactivity. This indicates that functionalized hydroxyapatite maintains the intrinsic chemical core required for expected performance in applications related to biological or material science.

#### XRD analysis

X-ray diffraction (XRD) was performed to determine the crystalline structure of fHAP and compare it with bHAP. The XRD patterns of both materials showed diffraction peaks indexed to some specific Miller indices, which correspond to the planes within the hydroxyapatite crystal lattice. The indexed planes in the fHAP are (002), (211), (310), (222), (213), and (004), and were also observed in bHAP. This similarity suggests that the crystal structure of hydroxyapatite was maintained after functionalization, preserving its structural integrity, which is crucial for applications such as bone tissue engineering or implant coating.

#### Raman spectroscopy analysis

Raman spectroscopy was used to analyze the vibrational modes of fHAP and bHAP. The Raman spectrum was dominated by several active vibrational modes, thereby providing insight into the molecular structure and composition of the samples. For fHAP, the activity of the Raman modes was observed at 437 cm^−1^, 965 cm^−1^, 1048 cm^−1^, 1085 cm^−1^, 1318 cm^−1^, 1428 cm^−1^, and 3577 cm^−1^, representing the vibrational behavior of phosphate and hydroxyl groups. The peak at 965 cm^−1^, corresponding to the symmetric stretching mode of phosphate groups, is the signature peak for hydroxyapatite. A comparison was made with bHAP, whose Raman modes are 435 cm^−1^, 592 cm^−1^, 963 cm^−1^, 1052 cm^−1^, 1075 cm^−1^, and 3579 cm^−1^. Following functionalization, their presence in both fHAP and bHAP would confirm the retention of hydroxyapatite›s primary characteristics, particularly of the phosphate and hydroxyl groups. The minimal shifts in peak positions between fHAP and bHAP, at around 1048 cm^−1^ and 1052 cm^−1^, suggests that the functionalization process caused minor alterations in the local environment of functional groups. However, the overall similarity in Raman spectra between fHAP and bHAP indicates that hydroxyapatite's core molecular structure remained intact, ensuring its bioactivity.

#### BET analysis

BET analysis was conducted to calculate the surface area, pore size, and pore volume of fHAP and compare it with bHAP. These parameters are important for applications such as catalysis, drug delivery, and bone tissue engineering, where surface area and porosity influence the material's interaction with its environment. The analysis revealed that fHAP had a surface area of 29.0862 m²/g, a pore size of 22 nm, and a pore volume of 0.162846 cm^3^/g. These values suggest that fHAP has a relatively high surface area and moderate pore size, which enhance its ability to interact with surroundings. In comparison, bHAP showed a higher surface area of 15.8382 m²/g, a greater pore size of 42 nm, and a slightly lower pore volume of 0.146834 cm^3^/g. The decrease in surface area for bHAP indicates that the functionalization process has significantly expanded the available surface area. The smaller pore size in fHAP, compared to bHAP, could indicate a more uniform or compact pore structure, which could be advantageous in applications requiring controlled diffusion or better interaction with smaller molecules.

### Histological examination

Analysis of the fourth-week group using H&E staining showed that mean NBF was 8009.620 μm^2^ in AG, 9256.200 μm2 in fHAP, and 3403.584 μm^2^ in the bHAP group Statistical analysis showed a significant difference between AG-bHAP (p<0.001) and fHAP-bHAP (p<0.001). No significant difference was found between AG-fHAP (p>0.05). (Table 1)

**Table 1:** Comparison of the results of H&E staining in the fourth-week group

Group	Median	25%	75%	p
AG	8009,62^a^	3976,136	19377,385	
fHAP	9256,2^a^	4752,3785	22825,923	<0.001*
bHAP	3403,584^b^	1902,2775	5440,7215	
*Kruskal-Wallis				
**Comparison**	**Diff of Ranks**		**q**	P<0.05*
AG - bHAP	3490,000		8,436	Yes
AG - fHAP	281,000		0,679	No
fHAP - bHAP	3209,000		7,756	Yes
* Tukey				

AG: Autogenous Graft, fHAP: Fish Derived Hydroxyapatite, bHAP: Bovine Derived Hydroxyapatite

**Figure 4:**
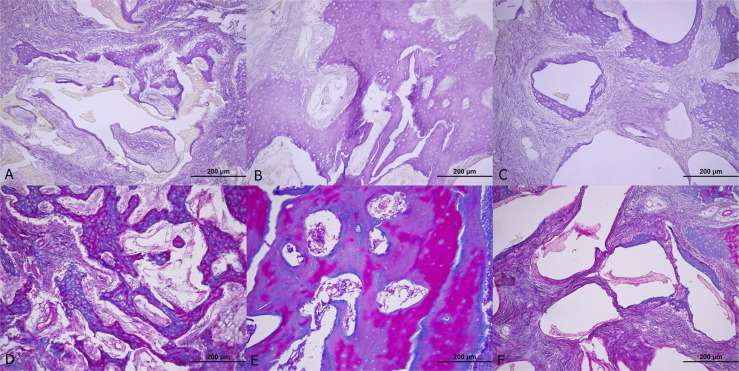
Histological examination of 4th week group. A, B and C H&E (hematoxylin and eosin) staining of fHAP, AG and bHAP, respectively. D, E and F Masson's trichrome staining of fHAP, AG and bHAP, respectively.

**Figure 5:**
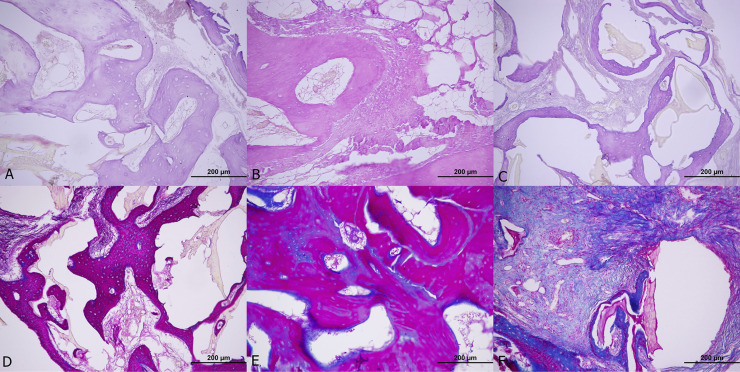
Histological examination of 8th week group. A, B and C C H&E (hematoxylin and eosin) staining of fHAP, AG and bHAP, respectively. D, E and F Masson's trichrome staining of fHAP, AG and bHAP, respectively.

The M&T staining analysis of the fourth-week group showed no significant difference. (p=0.058) (Figure 4). For the eighth-week group, H&E staining analysis showed a mean NBF of 5593.248 μm^2^ in AG, 5593.064 μm^2^ in fHAP, and 3988,392 μm^2^ in the bHAP group, with no statistical difference. (p=0.130). Similarly, the M&T staining analysis of the eighth-week group showed no significant difference. (p=0.331) (Figure 5)

Since histomorphometric measurements provided evidence of ossification (NBF) differences between the groups and were evaluated statistically, descriptive analyses were not used.

### Micro-CT examination

No statistically significant difference was found in terms of BMD, BV, TBV, TV, BV/TV, BS, BS/BV, BS/TV, TbTh, TbN, or TbSp between groups in the fourth-week group (p>0.05). However, in the eighth-week group, a statistically significant difference was found between fHAP and bHAP in BV. (p=0.007) (Table 2)

**Table 2:** Comparison of the Micro-CT results of the eighth-week group

Parameter	Group	Mean	SD	SE	p
BV (mm^3^)	AG^a^	8,2714	4,1081	2,0541	0.023*
	fHAP^a^	11,1595	7,9823	2,8222	
	bHAP^b^	2,7052	2,0129	0,7117	
	AG	110.19	23.889	11,9445	
BS-BV (%)	fHAP	79,7151	27,5683	9,7469	0.324
	bHAP	88,0844	38,8238	13,7263	
	AG	6,5459	1,4835	0,7418	
BS-TV (%)	fHAP	7,3231	2,8229	0,998	0.367
	bHAP	5,0423	3,9126	1,3833	
	AG	0,676	0,3579	0,1789	
Tb-Sp (mm)	fHAP	0,5764	0,2388	0,0844	0.767
	bHAP	0,6831	0,3531	0,1248	

* One-way Anova

AG: Autogenous Graft, fHAP: Fish Derived Hydroxyapatite, bHAP: Bovine Derived Hydroxyapatite BV: Bone Volume, BS-BV: New bone surface/Bone Volume, BS-TV: New bone surface/Tissue Volume, Tb-Sp: Trabecular Separation

## Discussion

This study aimed to evaluate the effect of fHAP on bone healing in an experimental sinus-lifting model in rabbits. The results showed no significant difference between the fHAP and AG groups regarding NBF and mineralized tissue (M&T) staining in both the fourth- and eight-week groups. However, there was a significant difference between the fHAP and bHAP groups in the fourth-week group regarding NBF, as well as a substantial difference between the fHAP and bHAP groups regarding BV in the eighth-week group.

Maxillary sinus augmentation with lateral window technique has become popular for achieving vertical bone height in the posterior maxilla to place dental implants of ideal length. Animal models have been used to investigate graft materials and surgical techniques. Yılmaz, et al.^9^ (2017) concluded that while the rabbit maxillary sinus augmentation model does not perfectly reflect human physiology, it has similar potential for demonstrating NBF histologically. Our study used a rabbit model to simulate human bone regeneration.

AG is considered the gold standard for bone grafting due to its ideal biological properties, including osteogenic, osteoinductive, and osteoconductive effects.^9–12^ However, AG has several other drawbacks, such as the need for a new surgical site, morbidity at the donor site, and a limited supply of graft material.^3^ The merits and limitations of AG are subject to ongoing debate. As a result, alternative grafting materials such as freeze-dried bone, demineralized freeze-dried bone, and xenogenic or synthetic bone substitutes have been used.^10^

Sununliganon, et al.^2^ (2014) investigated the effect of bone marrow concentrate, consisting of mesenchymal stem cells, on NBF in maxillary sinus lifting and concluded that bone marrow concentrates and bone marrow concentrate + bHAP showed better results for graft tissue/tissue volume. However, bone marrow harvesting for maxillary sinus lift is not a clinically practical method, and culturing undifferentiated mesenchymal stem cells is costly, limiting clinical applicability.

Xenografts have gained popularity as a solution to the limitations of AG.^10^ Xenografts offer excellent bioactivity, mechanical stability, angiogenic properties, and no toxicity, inflammatory, or antigenic reactions.^11,12^ Moreover, reductions in surgery time, cost, and morbidity rate make allogenic and xenogenic grafts appealing alternatives to AG.^3^ However, Prathibha, et al.^18^ (2024) found that the xenografts’ resorption pattern and integrity with the host may limit their potential. In our study, fHAP was compared with AG and bHAP, with fHAP showing a regeneration potential similar to AG and better than bHAP. This could be attributed to microstructure and chemical similarity between fHAP and AG. Sankar, et al.^25^ (2008) further reported that fHAP might contain trace elements such as chloride, fluoride, sodium and magnesium, which could promote bone regeneration.

Guided bone regeneration typically involves filling a defect with an appropriate graft material and covering it with a membrane. Collagen membranes, along with pericardium or polytetrafluoroethylene (PTFE) membranes, are preferred due to affordability and the fact that secondary surgery for removal is not needed. In this study, a resorbable collagen material was applied to all graft sites to avoid second surgery and minimize costs, while also avoiding any potential regenerative effects of the membrane itself. Tissue pressure to the surgical site may disrupt the graft healing, possibly leading to intervention of the mucosa. As such, reinforced membranes with titanium were used to overcome the flap pressure and to obtain the desired bone regeneration. Chierico, et al.^26^ (1999) showed that the titanium-reinforced GTAM membranes charged with negative electric field increased bone regeneration compared to neutral or positively charged membranes. Despite their regenerative potential, titanium-reinforced membranes are expensive, require a second surgery for removal, and can sometimes be exposed from the mucosa. Our study did not use titanium-reinforced membranes as the graft materials were stabilized within the sinus cavity, and no pressure on the graft was expected due to the flexibility of the rabbit skin.

Shi, et al.^14^ (2018) produced fHAP from three different fish species, characterizing the material through FT-IR, XRD, and SEM. As we followed their calcination protocol, the same character analysis methods were used to identify the fHAP. FT-IR, XRD, and SEM analyses confirmed that fHAP shares a similar chemical compound to bHAP, with greater surface area and volume, likely contributing to its NBF levels comparable to AG.

All xenografts (including fHAP) are based on calcium phosphate and consist of HAP, which closely resembles natural bone and provides osteoconductive properties.^9,10,17,19–23^ Granito, et al.^13^ (2018) reviewed the literature and concluded that fHAP is non-cytotoxic and suitable for biomedical applications. Venkatesan, et al. reported that fHAP positively interacted with mesenchymal stem cells (MSCs).^17^ Shi, et al.^14^ (2018) found a positive interaction between osteoblasts and fHAP, suggesting its potential use in bone repair or regeneration procedures. Yamamura, et al.^24^ (2018) confirmed the biocompatibility of fHAP in subcutaneous tissue tests on rats. Prathibha, et al.^18^ (2024) conducted an *in vivo* and *in vitro* study on fish scale-derived HAP and compared with commercial xenografts, reporting superior bone regeneration *in vivo* and positive interactions with mesenchymal stem cells *in vitro*; these results are consistent with ours, using fish spine-derived HAP. Additionally, we compared fish spine-derived HAP with AG, which was not the case for Prathibha, et al.^18^ (2014). To our knowledge, this is the first *in vivo* study comparing fish spine-derived HAP with AG, showing promising results on new bone formation.

The relatively small amounts of graft material used in our study may not fully replicate the natural process of the bone regeneration when compared to the quantities of external sinus lifting procedure in humans. Furthermore, the resorption percentage and *in vitro* cell interaction of the fish spine-derived HAP were not evaluated, as these were planned for a separate study. These can be listed as the limitations of present study.

## Conclusion

This study demonstrated that fHAP has a similar effect on bone healing and regeneration as AG and performs better than bHAP in an experimental sinus-lifting model. Given its excellent bioactivity and mechanical stability, angiogenic properties, and absence of toxicity, inflammatory, and antigenic reactions, fHAP can be considered as an alternative to AG as a bone substitute. However, further studies are needed to investigate the effects of fHAP on bone healing in other animal models and in clinical trials with humans.

## Data Availability

All data generated or analyzed during this study are included in this published article
