# ERRATUM

**DOI:** 10.1590/1678-7757-2024er002

**Published:** 2025-02-21

Due to a publishing error the article: “**Fish: a new xenograft source for maxillary sinus lifting**”, published at Journal of Applied Oral Science 32(e20240245):1-11. doi: 10.1590/1678-7757-2024-0245 was published with the following error:

Where it reads:

**Authors**

0000-0002-9828-5096 Emrah SOYLU^1^

Musab Süleyman KILAVUZ^1^

Fatih DUMAN^2^

Hasan EKEER^3^

Zeynep Burçin GÖNEN^1^

Beyza KAHRAMAN^1^

Arzu Hanım YAY^4^

Demet BOLAT^4^

^1^Erciyes University Faculty of Dentistry, Department of Oral and Maxillofacial Surgery and DentBioChem Biotechnology Co., Erciyes Teknopark, Kayseri, Türkiye.

^2^Erciyes University, Faculty of Science, Department of Hydrobiology, Kayseri, Türkiye.

^3^Biologist, Erciyes University, Faculty of Dentistry, Research Laboratory, Kayseri, Türkiye.

^4^Erciyes University, Faculty of Medicine, Department of Histology and Embryology, Kayseri, Türkiye.

It should read:

**Authors**

0000-0002-9828-5096 Emrah SOYLU^1,2^

Musab Süleyman KILAVUZ^1^

Fatih DUMAN^3^

Hasan EKEER^4^

Zeynep Burçin GÖNEN^1^

Beyza KAHRAMAN^1,5^

Arzu Hanım YAY^6^

Demet BOLAT^6^

^1^Erciyes University Faculty of Dentistry, Department of Oral and Maxillofacial Surgery, Kayseri, Turkey.

^2^DentBioChem Biotechnology Inc., Erciyes Teknopark, Kayseri, Turkey.

^3^Erciyes University, Faculty of Science, Department of Hydrobiology, Kayseri, Turkey.

^4^Erciyes University, Faculty of Dentistry, Research Laboratory, Kayseri, Turkey.

^5^Erciyes University Institute of Health Sciences, Department of Oral and Maxillofacial Surgery, Kayseri, Turkey.

^6^Erciyes University. Faculty of Medicine, Department of Histology and Embryology, Kayseri, Turkey.

